# A real-world study of different doses of rivaroxaban in patients with nonvalvular atrial fibrillation

**DOI:** 10.1097/MD.0000000000038053

**Published:** 2024-04-26

**Authors:** Xinsheng Yan, Litao Zhang, Dan Zhang, Xiaosu Wang

**Affiliations:** a Department of Clinical Laboratory, Wuhan Asia General Hospital, Wuhan Asia General Hospital Affiliated to Wuhan University of Science and Technology, Wuhan, Hubei Province, People’s Republic of China.

**Keywords:** atrial fibrillation, dose, effectiveness, rivaroxaban, safety

## Abstract

To explore the anticoagulant effect and safety of utilizing different doses of rivaroxaban for the treatment of patients with atrial fibrillation (AF) in the real world. A retrospective case-control analysis was performed by applying the hospital database, and 3595 patients with non-valvular atrial fibrillation (NVAF) who were hospitalized and taking rivaroxaban at Wuhan Asia Heart Hospital and Wuhan Asia General Hospital from March 2018 to December 2021 were included in the study, and were divided into the rivaroxaban 10 mg and 15 mg groups according to the daily prescribed dose, of which 443 cases were in the 10 mg group and 3152 cases were in the 15 mg group. The patients were followed up regularly, and the incidence of thrombotic events, bleeding events and all-cause deaths were recorded and compared between the 2 groups, and logistic regression was applied to analyze the influencing factors for the occurrence of adverse events. Comparison of the incidence of thrombosis, bleeding and all-cause death between the 2 groups of patients showed that the 10 mg group was higher than the 15 mg group, but the difference was not statistically significant (*χ*^2^ = 0.36, 3.26, 1.99, all *P *> .05); the incidence of total adverse events between the 2 groups of patients was higher in the 10 mg group than in the 15 mg group, with a statistically significant difference (*χ*^2^ = 4.53, *P *= .033); multifactorial logistic regression results showed that age [OR (95% CI) = 1.02 (1.00–1.04)], diabetes mellitus [OR (95% CI) = 1.69 (1.09–2.62)], D-dimer level [OR (95% CI) = 1.06 (1.00–1.11)] and persistent AF [OR (95% CI) = 1.54 (1.03–2.31)] were risk factors for adverse events (*P* < .05). In the real world, Asian clinicians recommend rivaroxaban 10 mg once daily for NVAF patients for a variety of reasons; however, this dose is not superior or even inferior to the 15 mg group in terms of effectiveness and safety. Advanced age, elevated D-dimer levels, history of diabetes mellitus, and persistent AF are risk factors for adverse events, and the optimal dosage of rivaroxaban or optimal anticoagulation strategy for Asian patients with nonvalvular AF requires further study.

## 1. Introduction

Atrial fibrillation (AF) significantly increases the risk of stroke, and anticoagulation is an important component of comprehensive AF management.^[1]^ In recent years, direct oral anticoagulants (DOAC´s) have gradually replaced warfarin as the guideline-recommended anticoagulant of choice for the prevention of stroke and systemic embolism in patients with nonvalvular atrial fibrillation (NVAF).^[2]^ Rivaroxaban, as the first oral direct factor Xa inhibitor, is clinically favored because it has an anticoagulant effect that is not inferior to that of warfarin^[3]^ and does not require routine monitoring of the international normalized ratio and prothrombin time (PT). However, due to the inter-individual variability of pharmacokinetics, insufficient anticoagulation or excessive anticoagulation may still occur, especially as the clinical application of rivaroxaban becomes more and more widespread, and the safety issue in emergencies or special circumstances has gradually attracted clinical attention.^[4]^ Many physicians tend to use smaller doses to reduce the risk of bleeding, which in turn often results in insufficient anticoagulation, and these problems plague physicians and largely affect the rational use of the drug. Although both the European Society of Cardiology (ESC) guidelines^[2]^ and the American College of Cardiology guidelines^[5]^ recommend 20 mg/d as a stroke-prevention dose for nonvalvular NVAF, fewer patients were given the 20 mg dose due to physicians’ concerns about bleeding, so the 15 mg/d and 10 mg/d doses have been commonly used in Asian patients with NVAF.^[6,7]^ There is a lack of data from studies of rivaroxaban in Asian populations, and the clinical benefits and risks of different dosages in patients with AF and the choice of anticoagulation strategy in the presence of different complications in patients remain to be addressed. The aim of this study was to investigate the effectiveness and safety of rivaroxaban at 10 mg dose and 15 mg dose for the treatment of AF in the real world, with the aim of informing the use of rivaroxaban for anticoagulation in patients with AF.

## 2. Materials and methods

### 2.1. Study subjects

Patients with AF admitted to Wuhan Asia Heart Hospital and Wuhan Asia General Hospital from March 2018 to December 2021 were selected as the study subjects, and the International Classification of Diseases code was used as the search condition to retrieve patients with discharge diagnosis of AF and flutter (International Classification of Diseases code I48). Inclusion criteria: the primary reason for the hospitalization was AF, the diagnosis of AF was based on electrocardiographic evidence and anticoagulation with rivaroxaban. Exclusion criteria: history of cardiac valvuloplasty or replacement; malignant neoplastic disease; change of rivaroxaban medication; and incomplete medical records. The study finally included 3595 patients who met the inclusion and exclusion criteria, 2172 males (60.4%) and 1423 females (39.6%), with ages ranging from 20 to 91, and a median age of 65 years. 443 patients (12.3%) were enrolled in the 10 mg group, and 3152 patients (87.7%) were enrolled in the 15 mg group.

### 2.2. Methods

This study is an observational study, according to the patients’ anticoagulant therapy using rivaroxaban dose is divided into 10 mg group (10 mg/d) and 15 mg group (15 mg/d), 10 mg group patients orally rivaroxaban tablets (Bayer Pharmaceuticals & Healthcare Ltd.) 10 mg once a day, 15 mg group patients orally rivaroxaban tablets (Bayer Pharmaceuticals & Healthcare Ltd.) 15 mg orally once a day. Patients were maintained on anticoagulation therapy until adverse events occurred or until they were switched to another anticoagulant for economic or availability reasons. The effects were followed up and observed from the beginning of treatment. The baseline clinical data of the patients were collected through the electronic medical record system, including age, gender, body mass index (BMI), CHA2DS2-VASc score, HAS-BLED score, underlying diseases (hypertension, diabetes mellitus, etc), and the use of comorbid medications (aspirin). The results of the relevant laboratory tests were also recorded, such as D-dimer, liver function, renal function, cardiac function and other results. Outpatient or telephone follow-up was used to record and compare the incidence of thrombosis, bleeding and death between the 2 groups during the follow-up period. Risk factors for adverse events were analyzed using multifactorial logistic regression. The study was ethically reviewed by the participating medical institutions, and the ethical number of Wuhan Asia General Hospital was WAGHMEC-KT-2021009, and the ethical number of Wuhan Asia Heart Hospital was 2023-B017.

### 2.3. Follow-up

The follow-up time was started from the patients’ initial discharge, with a maximum of 2 years and a mean follow-up time of 18 months. The patients’ first hospitalization electronic medical record information was regarded as the baseline record, and the validity endpoint events included thrombotic events, hemorrhagic events, and all-cause death. Embolic events included stroke, myocardial infarction, pulmonary embolism, arterial embolism, venous embolism, atrial thrombosis, auricular thrombosis, and transient ischemia attack; and hemorrhagic events included cerebral hemorrhage, gastrointestinal hemorrhage, urethral hemorrhage, and others.

### 2.4. Statistical analysis

Statistical analysis was performed with MedCalc 16.2.1 software. Normally distributed continuous variables were expressed as mean and standard deviation (mean ± SD), and the *t* test was used for intergroup comparisons; non-normally distributed continuous variables were expressed as median and interquartile range, and the Mann-Whitney test was used for intergroup comparisons; categorical variables were described by frequency and percentage [n(%)], and intergroup comparisons were performed by the Pearson Chi-square test or Fisher exact test; risk factors for adverse events were analyzed using multifactorial logistic regression, and odds ratios (OR) and 95% confidence intervals (CI) were calculated. The statistical significance level was defined as *P* < .05.

## 3. Results

### 3.1. Comparison of clinical baseline data

Comparison of the general data between the 2 groups showed that the differences in age, gender, BMI, hypertension, diabetes mellitus, renal function, cardiac function, D-dimer, comorbid acute coronary syndrome (ACS), coadministration of aspirin, CHA2DS2-VASc scores, and HAS-BLED scores were statistically significant, as shown in Table 1.

**Table 1:** Comparison of general information in different dose groups of rivaroxaban.

Characteristics	10 mg group (n = 443)	15 mg group (n = 3152)	*P* value
Age (yr)	72 (66, 78)	65 (57, 70)	<.001
Female	219 (49.4)	1204 (38.1)	<.001
BMI (kg/m^2^)	24.40 ± 3.60	25.17 ± 3.45	<.001
Hypertension	303 (68.3)	1844 (58.5)	<.001
Diabetes mellitus	116 (26.1)	605 (19.1)	<.001
Renal dysfunction	95 (21.4)	196 (6.2)	<.001
Cardiac insufficiency	201 (45.3)	762 (24.1)	<.001
History of stroke	169 (38.1)	1080 (34.2)	.108
Comorbid ACS	171 (38.6)	543 (17.2)	<.001
Co-administration of aspirin	203 (45.8)	819 (25.9)	<.001
History of smoking	138 (31.1)	1010 (32.0)	.706
History of alcohol consumption	70 (15.8)	575 (18.2)	.210
D-dimer (μg/mL)	0.87 ± 1.89	0.53 ± 1.72	<.001
Persistent atrial fibrillation	241 (54.4)	1588 (50.3)	.113
CHA_2_DS_2_-VAS_C_ score	3.80 ± 1.83	2.79 ± 1.79	<.001
HAS-BLED score	2.08 ± 1.22	1.47 ± 1.15	<.001

Values are expressed in n (%) or mean ± SD or median (IQR).

ACS = comorbid acute coronary syndrome, BMI = body mass index, CHA2DS2-VASc score = Congestive Heart Failure, Hypertension, Age ≥ 75 [Doubled], Diabetes Mellitus, Prior Stroke or Transient Ischemic Attack [Doubled], Vascular Disease, Age 65–74, Female, HAS-BLED score = Hypertension, Abnormal renal/liver function, Stroke, Bleeding history or predisposition, Labile INR, Elderly (>65), Drugs/alcohol concomitantly.

### 3.2. Comparison of the incidence of adverse events

During the follow-up period, 7 cases (1.58%) of thrombotic events, 9 cases (2.03%) of hemorrhagic events, and 4 cases (0.90%) of all-cause deaths occurred in the 10 mg group, by comparison, 39 cases (1.24%) of thrombotic events, 33 cases (1.05%) of hemorrhagic events, and 13 cases (0.41%) of all-cause deaths occurred in the 15 mg group, and the differences were not statistically significant (*P* > .05); Comparison of the incidence of total adverse events between the 2 groups of patients showed that 20 cases (4.51%) occurred in the 10 mg group was significantly higher than 85 cases (2.70%) in the 15 mg group, and the difference was statistically significant (*χ*^2^ = 4.53, *P* = .033). See Table 2.

**Table 2:** Comparison of endpoint events in patients in different dose groups of rivaroxaban [*n* (%)].

Events	10 mg group (n = 443)	15 mg group (n = 3152)	*χ* ^2^	*P* value
Thrombotic events	7 (1.58)	39 (1.24)	0.36	.548
Ischemic stroke	3 (0.68)	25 (0.79)	0.07	.792
Systemic embolism	3 (0.68)	9 (0.29)	1.79	.180
Myocardial infarction	1 (0.23)	5 (0.16)	0.11	.746
Bleeding events	9 (2.03)	33 (1.05)	3.26	.071
Cerebral hemorrhage	1 (0.23)	3 (0.10)	0.60	.440
Gastrointestinal bleeding	1 (0.23)	5 (0.12)	0.11	.746
Urinary tract bleeding	4 (0.90)	18 (0.57)	0.70	.402
Others	1 (0.23)	4 (0.13)	0.27	.601
Hemoglobin drop ≥ 20g/L	2 (0.45)	3 (0.10)	3.55	.060
All-cause death	4 (0.90)	13 (0.41)	1.99	.159
Cardiac death	2 (0.45)	7 (0.22)	0.82	.366
Death from lung infection	0 (0.00)	2 (0.06)	0.28	.596
Cancer death	0 (0.00)	2 (0.06)	0.28	.596
Unexplained deaths	2 (0.45)	2 (0.06)	5.26	.022
Total	20 (4.51)	85 (2.70)	4.53	.033

### 3.3. Analysis of factors influencing the occurrence of adverse events in patients

A multifactorial regression analysis of age, gender, BMI, rivaroxaban dose, hypertension, diabetes mellitus, renal dysfunction, cardiac insufficiency, history of stroke, comorbid ACS, co-administration of aspirin, history of smoking, history of alcohol consumption, D-dimer level, and persistent AF of each of the clinical factors on the adverse events, including thrombotic events, hemorrhagic events, and all-cause mortality, revealed that age [OR (95% CI) = 1.02 (1.00–1.04)], diabetes mellitus [OR (95% CI) = 1.69 (1.09–2.62)], D-dimer level [OR (95% CI) = 1.06 (1.00–1.11)], and persistent AF [OR (95% CI) = 1.54 (1.03–2.31)] were independent risk factors (all *P *< .05). See Table 3.

**Table 3:** Multifactorial logistic regression analysis of adverse events.

Factors	OR	95%CI	*P* value
Age	1.02	1.00–1.04	.025
Diabetes	1.69	1.09–2.62	.018
Co-administration of aspirin	0.67	0.42–1.07	.096
D-dimer levels	1.06	1.00–1.11	.042
Persistent atrial fibrillation	1.54	1.03–2.31	.036

Factors included in the regression analysis included age, gender, body mass index, rivaroxaban dose, hypertension, diabetes mellitus, renal dysfunction, cardiac insufficiency, history of stroke, comorbid acute coronary syndrome, co-administration of aspirin, history of smoking, history of alcohol consumption, D-dimer level, and persistent atrial fibrillation.

CI = confidence interval, OR = odds ratios.

## 4. Discussion

The main finding of this study is that for patients with NVAF who were anticoagulated with DOAC´s in the Chinese population, patients in the rivaroxaban 10 mg dose group did not have a significant benefit, and were not superior and even showed inferior performance of the 10 mg dose than the 15 mg dose group in terms of effectiveness and safety. This finding provides a reference for evaluating the optimal dose of DOAC´s for use in Asian populations.

The results of our study also showed that in actual clinical practice a significant proportion of patients used rivaroxaban 15 mg/d as a treatment for stroke prevention, with a portion using the 10 mg/d dose and a smaller proportion of other doses (data not provided), which seems to be inconsistent with the recommended dose of the guideline,^[8]^ which is mainly due to the fact that in actual clinical practice, bleeding was found to be a higher risk in patients using the 20 mg/d regimen. The applicability of the standardized dose of 20 mg/d recommended in the rivaroxaban insert to Asian populations is debated. It has been shown that Asian patients with AF have a higher risk of cerebral hemorrhage compared to non-Asians treated with DOAC´s.^[9]^ Ethnic differences between Asian populations and European and American populations may appear to have different pharmacokinetic effects, and there is a lack of large-scale prospective randomized controlled trials in Asia assessing the effectiveness and safety of different doses of rivaroxaban for stroke prevention in AF,^[10]^ and the randomized controlled trials that have tested these medications for the prevention of stroke in AF have tested only a few patients at reduced doses.^[11]^ Due to a pharmacokinetic profile that differs from that of the Caucasian population, the dose of rivaroxaban approved in Japan is lower than that generally recommended in other countries or regions, and the results of a Japanese Rivaroxaban Once Daily Oral Direct Factor Xa Inhibition Compared with Vitamin K Antagonism for Prevention of Stroke and Embolism Trial in Atrial Fibrillation study in Japan showed that the optimal dose of rivaroxaban to be used in patients with nonvalvular atrial fibrillation was 15 mg/d, which had a clinical benefit similar to that of 20 mg/d in Caucasians.^[12]^ In addition, a rivaroxaban dose of 10 mg/d has been widely used in Asian countries.^[13,14]^

In our analysis, we found that patients in the 10 mg group had a median age of 72 years, which was significantly higher than that of 65 years in the 15 mg group (*P* < .001), and more patients with renal dysfunction (21.4% vs 6.2%), which is in line with the recommendation of dose reduction in patients with advanced age as well as renal impairment, compared to patients using the 15 mg dose of rivaroxaban; furthermore, in the 10 mg group women (49.4% vs 38.1%), low body weight (24.40 ± 3.60 vs 25.17 ± 3.45), and cardiac insufficiency (45.3% vs 24.1%) accounted for significantly more patients in the 10 mg dose group than in the 15 mg dose group and combined with more underlying conditions such as hypertension (68.3% vs 58.5%) and diabetes mellitus (26.1% vs 19.1%), and more patients co-administered with the antiplatelet drug (aspirin) (45.8% vs 25.9%), which was mainly used to prevent ischemic heart disease. All of which can exacerbate clinicians’ concerns about bleeding risk, leading to lower dose selection. In a study investigating the reasons for underdosing in prescribing, older age, female gender, low body weight, high serum creatinine levels, or comorbidities of congestive heart failure or vascular disease were shown to be factors associated with underdosing descriptions,^[15]^ which is consistent with the results of this study.

In this study, it was found that the 10 mg group was associated with poorer outcomes. The incidence of total adverse events was higher in the 10 mg group than in the 15 mg group, and the difference was statistically significant (*P* = .033). The incidence of thrombotic events and all-cause mortality was higher in the 10 mg group than in the 15 mg group, but the difference was not statistically significant. For bleeding events, although not statistically significant (*P* = .071), there was a trend towards an increase in the incidence of bleeding events in the 10 mg group, which is not consistent with the general perception, but the cause of the inconsistency may be related to the fact that patients in the 10 mg group patients had a higher baseline risk of bleeding. The results of the HAS-BLED score showed that the score of the 10 mg group was significantly higher than that of the 15 mg group (*P* < .001), suggesting that patients in the 10 mg group may have a higher risk of bleeding, and that physicians may choose a lower dose because of the fear of bleeding when making anticoagulation decisions. However, the findings also showed that the CHA2DS2-VASc score was also higher in the 10 mg group than in the 15 mg group, suggesting that multiple confounding factors often exist in clinical practice, and that anticoagulation in AF needs to be based on the individualized judgment of clinicians.^[16]^ In a real-world study of Asian patients with NVAF, a study from Taiwan Province of China also compared the effectiveness and safety of 10 mg/d (low-dose) rivaroxaban with 15 and 20 mg/d (standard-dose) rivaroxaban in an Asian AF population, and demonstrated that the risk of infarction was higher for low-dose rivaroxaban, while the risks of embolism and bleeding were similar in the Asian AF patient population.^[17]^ Another study in Taiwan showed that after lowering the dose of the drug, not only did it fail to achieve an anticoagulant effect, but it also failed to even reduce the risk of bleeding.^[18]^ An international XANTUS study also showed that patients treated with non-recommended doses of rivaroxaban had a higher risk of major bleeding, stroke/non-central nervous system disease, and all-cause mortality than those who received the recommended dose, and these differences were no longer significant after adjusting for the baseline characteristics of the patients who received the non-recommended dose.^[19]^ Multiple other reports have shown that underdosing shows worse outcomes in stroke prevention and no benefit for bleeding complications.^[20,21]^ A meta-analysis, however, noted that inadequate over-the-counter dosing of rivaroxaban was associated with an increased risk of stroke or system-wide embolization (HR = 1.31, 95% CI 1.05–1.63; *P* = .02).^[22]^ The absorption, metabolism, and excretion processes of rivaroxaban involve several enzymes and are regulated by a variety of genes, including ABCB1, ABCG2, CYP3A4, CYP3A5, and CYP2J2. A multicenter prospective cohort study identified genetic variants affecting bleeding risk and rivaroxaban pharmacodynamics in patients with NVAF, and at 12-month follow-up, the SUSD3 rs76292544 mutant TT genotype significantly increased the risk of rivaroxaban bleeding (32.9% to 100%), compared with TT and TC carriers of NCMAP rs4553122, CC carriers had a 20.8% higher peak level of rivaroxaban anti-FXa, and bleeding events were significantly associated with peak anti-FXa levels (*P *= .027).^[23]^ Therefore, it is reasonable to hypothesize that the occurrence of adverse events may be related to the patient drug concentration levels, baseline levels, and genetic polymorphisms rather than the dose itself, but further studies are needed to confirm this. There are many variables but none of them seem to explain this “paradoxical” behavior concerning the dosage, therefore, these results pose more questions for further studies.

Further analysis in this study revealed that D-dimer level was a risk factor for the occurrence of adverse events, a result consistent with previous studies, mainly because D-dimer is a highly sensitive biomarker of thrombosis,^[24]^ and high D-dimer levels are associated with an increased incidence of cardiovascular events,^[25]^ and D-dimer can be used as a prognostic marker for prognosis evaluation of anticoagulant therapy in patients with AF, whereas the question of whether D-dimer can be used to guide anticoagulation therapy in AF patients requires further study. The results of this study also showed that advanced age, history of diabetes mellitus, and persistent AF were also risk factors for adverse events in patients with AF, and these factors were confirmed in several studies.^[26,27]^ In addition, no statistically significant correlation between rivaroxaban dose and adverse events was observed in the logistic regression analysis of this study, which may further suggest that the difference in the occurrence of adverse events between patients treated with the 10 mg dose and the 15 mg dose of rivaroxaban may not be significantly related to the rivaroxaban dose itself.

## 5. Study limitations

Firstly, the study was a retrospective analysis, and the included population was from Wuhan, China, which may be subject to selection bias, and the applicability of the findings to other regions needs to be further evaluated due to potential population differences and differences in the level of medical care in different regions. Secondly, there were other unmeasured factors during the course of this study, such as use of other anticoagulant medications, medications, metabolic gene polymorphisms, and drug reaction patterns. metabolic gene polymorphisms, and differences in drug responsiveness, which may also have a potential impact on the study conclusions and may provide a more in-depth analysis if this information is available. These limitations may inform the design of more prospective, multicenter randomized controlled studies in the future.

## 6. Conclusion

In summary, in the real world, Asian clinicians recommend rivaroxaban 10 mg once daily for patients with NVAF for a variety of reasons; however, this dose is associated with a higher incidence of adverse events, and is not superior or even inferior to the 15 mg group in terms of effectiveness and safety. Further prospective studies are needed to determine the optimal dose of rivaroxaban or the optimal anticoagulation strategy for the Asian NVAF population.

## Acknowledgments

The authors thank all the clinical staff contributed to the study.

## Author contributions

**Data curation:** Xinsheng Yan, Litao Zhang, Dan Zhang.

**Methodology:** Xiaosu Wang.

**Writing – original draft:** Xinsheng Yan, Xiaosu Wang.

**Writing – review & editing:** Xiaosu Wang.
